# Licochalcone A induces autophagy through PI3K/Akt/mTOR inactivation and autophagy suppression enhances Licochalcone A-induced apoptosis of human cervical cancer cells

**DOI:** 10.18632/oncotarget.4767

**Published:** 2015-07-31

**Authors:** Jen-Pi Tsai, Chien-Hsing Lee, Tsung-Ho Ying, Chu-Liang Lin, Chia-Liang Lin, Jung-Tsung Hsueh, Yi-Hsien Hsieh

**Affiliations:** ^1^ Department of Nephrology, Buddhist Dalin Tzu Chi General Hospital, Chiayi, Taiwan; ^2^ School of Medicine, Tzu Chi University, Hualien, Taiwan; ^3^ Graduate Institute of Medical Sciences, Chang Jung Christian University, Tainan, Taiwan; ^4^ Division of Pediatric Surgery, Department of Surgery, Children's Hospital of China Medical University, Taichung, Taiwan; ^5^ Department of Obstetrics and Gynecology, School of Medicine, College of Medicine, Chung Shan Medical University, Taichung, Taiwan; ^6^ Institute of Biochemistry, Microbiology and Immunology, Chung Shan Medical University, Taichung, Taiwan; ^7^ Department of Biochemistry, School of Medicine, Chung Shan Medical University, Taichung, Taiwan; ^8^ Clinical laboratory, Chung Shan Medical University Hospital, Taichung, Taiwan

**Keywords:** Licochalcone A, apoptosis, autophagy, cervical cancer

## Abstract

The use of dietary bioactive compounds in chemoprevention can potentially reverse, suppress, or even prevent cancer progression. However, the effects of licochalcone A (LicA) on apoptosis and autophagy in cervical cancer cells have not yet been clearly elucidated. In this study, LicA treatment was found to significantly induce the apoptotic and autophagic capacities of cervical cancer cells *in vitro* and *in vivo*. MTT assay results showed dose- and time-dependent cytotoxicity in four cervical cancer cell lines treated with LicA. We found that LicA induced mitochondria-dependent apoptosis in SiHa cells, with decreasing Bcl-2 expression. LicA also induced autophagy effects were examined by identifying accumulation of Atg5, Atg7, Atg12 and microtubule-associated protein 1 light chain 3 (LC3)-II. Treatment with autophagy-specific inhibitors (3-methyladenine and bafilomycin A1) enhanced LicA-induced apoptosis. In addition, we suggested the inhibition of phosphatidylinositol 3-kinase (PI3K)/Akt/mammalian target of mTOR pathway by LicA. Furthermore, the inhibition of PI3K/Akt by LY294002/si-Akt or of mTOR by rapamycin augmented LicA-induced apoptosis and autophagy. Finally, the *in vivo* mice bearing a SiHa xenograft, LicA dosed at 10 or 20 mg/kg significantly inhibited tumor growth. Our findings demonstrate the chemotherapeutic potential of LicA for treatment of human cervical cancer.

## INTRODUCTION

Cervical cancer is the fourth most common cancer in women, and the seventh most common type of cancer overall, with an estimated 528,000 new cases worldwide in 2012. There were an estimated 266,000 deaths from cervical cancer in 2012, accounting for 7.5% of all female cancer deaths [1]. Because of widespread screening programs coupled with advanced medical treatment technologies, women with cervical cancer have relatively high five-year survival rates, and there is a consensus that the early detection of cervical cancer can help prevent premature mortality [2]. Although human papillomavirus (HPV) is the major etiological agent of cervical cancer, HPV infection alone does not sufficiently explain the progression of cervical cancer. The suboptimal prognosis of patients with cervical cancer might be due to advanced or metastatic diseases with poor responses to therapies [3].

Apoptosis is an active process in which apoptotic cells undergo chromatin condensation and fragmentation followed by the formation of apoptotic bodies containing intact cytoplasmic organelles or fragments of the nucleus, resulting in a programmed and very regulated form of cell death executed by caspases and Bcl-2 family proteins [4]. Autophagy is an intracellular degradative system that plays important roles in regulating protein homeostasis, and is essential for survival when cells are faced with metabolic stress [5, 6]. Accumulating evidence suggests that cancer cells tend to have reduced autophagy relative to their normal counterparts and to premalignant lesions [7]. Several molecular and cell-signaling pathways have been implicated in regulating autophagy, such as the autophagy-related gene family [8], Beclin-1 [9], mitogen-activated kinase (MAPK) [10], and the phosphatidylinositol 3-kinase/AKT/mammalian target of rapamycin (PI3K-AKT-mTOR) pathways [11] Several pieces of evidence have also suggested that autophagy triggers tumor cell death in response to various anti-tumor agents [12, 13]. In contrast, autophagy induced by chemotherapy or radiotherapy may prevent cells from undergoing apoptosis, producing unfavorable conditions after anti-tumor therapy [14]. Thus, autophagy is also implicated in the promotion of cellular self-destruction, presumably due to the induction of mitochondria-dependent or -independent death signaling pathways, or to the irreversible destruction of cellular contents [15, 16]. However, the detailed mechanisms of different anticancer drug treatments, all of which necessarily involve the relationship between autophagy and apoptosis to some degree, are still poorly understood.

Licochalcone A (LicA) is a characteristic chalcone of licorice, which is the root of Glycyrrhiza inflata Batalin [17]. It is the most potent component of licorice and has been shown to have anti-inflammatory and anti-microbial activities [18], as well as anti-tumor effects [19,20]. LicA can induce prostate cancer apoptosis through the modulation of bcl-2 protein expression [19], suppress the migration of endothelial cells and the proliferation of smooth muscle by decreasing ERK1/2 activities and Rb phosphorylation, and block the progression of the cell cycle [21]. Moreover, mice fed with LicA exhibit significantly reduced tumor formation as well as reductions in the number of cells expressing proliferating cell nuclear antigen, beta-catenin, COX-2, and iNOS in the colon; LicA also significantly increases survival and inhibits liver metastasis, as well as the expression of matrix metalloproteinase-9 in the liver [20]. Recently, we also found that LicA could inhibit the invasion and migration of HCC cell lines via inhibition of the expression of uPA via the MKK4/JNK pathway [22]. Recent reports suggest that natural compounds, such as curcumin, resveratrol, oridonin, and quercetin, may induce cancer cell death by activating core autophagic pathways [23]. To our knowledge, only one article has been published up to now regarding the ability of LicA to induce caspase-dependent and autophagy-related cell death in prostate cancer LNCaP cells [24]. Hence, alternative cell death pathways (such as autophagic cell death) are of interest for drug development and cancer chemotherapy [25]. However, there is limited evidence for the effects of LicA on the underlying mechanisms in human cervical cancer cells.

The aim of the present study was to clarify the mode of action of LicA in the context of its anti-tumor activity, and to investigate the relationship between apoptosis and autophagy both *in vitro* and *in vivo*. Our data show that LicA treatment inhibited the growth of human cervical cancer cells. We also sought to identify the molecular mechanisms underlying the effects of LicA on apoptosis and autophagy. The results demonstrated that LicA induces caspase-dependent apoptosis and that LicA-induced early autophagy is mainly dependent on the induction of autophagosomes; the conversion of LC3-I to II; the induction of Atg5, Atg7, Atg12 and Beclin1; and the inhibition of Bcl-2. The data indicate that LicA ultimately induces apoptosis through the inhibition of the PI3K/Akt/mTOR pathway and the activation of caspases. We also found that LicA-induced apoptosis is enhanced by Akt inhibitor, siRNA-Akt, and rapamycin (mTOR inhibitor). Taken together, our observations suggest that LicA should be regarded as a promising anti-cancer agent against cervical cancer cells, and that the inhibition of PI3K/Akt/mTOR-mediated autophagy may enhance the anti-tumor effects of LicA, such that this inhibition may have therapeutic implications in the treatment of cervical cancer.

## MATERIALS AND METHODS

### Reagents and chemicals

Licochalcone A (LicA) was purchased from Sigma-Aldrich (St. Louis, MO, USA). The following antibodies were used in this research: anti-Bcl-2, anti-p-JNK, anti-JNK, anti-p-ERK1/2, anti-ERK1/2, anti-p-p38, anti-p38, anti-p-Akt, anti-Akt, and anti-β-actin were purchased from Santa Cruz (CA, USA). siRNA-Beclin1, siRNA-Akt, siRNA-Atg12 were purchased from Santa Cruz (CA, USA). The anti-cleaved-caspase-3, anti-cleaved-caspase-9, and anti-cleaved-PARP, mTOR Pathway and Autophagy Antibody Sampler Kit were purchased from Cell Signal Technology. Acidic vesicular organelle was purchased from AAT Bioquest, Inc. Annexin V-FITC and propidiumiodide (PI) kit was purchased from BD Biosciences (San Diego, CA). Z-VAD-FMK was purchased from BioVision. Bafilomycin A1 was purchased from Enzo Life Sciences. LY294002 and Rapmycin were purchased from Calbiochem. pBABEpuro GFP-LC3 was a gift from Jayanta Debnath (Addgene plasmid # 22405).

### Cell culture

Human cervical cancer cell lines and normal cell lines, SiHa (ATCC HTB35) was obtained from the American Type Culture Collection (ATCC, Rockville, MD), C33A (BCRC No.60554), HeLa (BCRC No 60005) and CaSki (BCRC No 60251), HK-2 (BCRC No 60097) and WI-38 (BCRC No 60504) were obtained from the Bioresources Collection and Research Center, Food Industry Research and Development Institute (Hsinchu, Taiwan). SiHa and HeLa cells were maintained in Dulbecco's modified Eagle's medium (DMEM, Gibco-Invitrogen Corporation, CA), C33A and WI-38 cells was maintained in modified Eagle's medium (MEM, Gibco-Invitrogen Corporation, CA), and CaSki cells was maintained in RPMI medium (Gibco-Invitrogen Corporation, CA), HK-2 was maintained in DMEM-F12 medium (Gibco-Invitrogen Corporation, CA), all cells were supplemented with 10 % fetal bovine serum (FBS, Gibco-Invitrogen Corporation, CA) and 1 % antibiotics (10,000 units/mL penicillin, 10 μg/mL streptomycin (Invitrogen Life Technologies, Carlsbad, CA) in a humidified atmosphere of 5 % CO_2_ at 37°C.

### Cell viability assay

The cytotoxic activity of LicA was measured using the MTT assay. SiHa and HeLa cells were treated with LicA at various concentrations (0, 10, 30, and 50 μM) for various periods of time (24 and 48 hours), and then washed once and incubated with MTT (0.5 mg/mL) at 37°C for 4 hours. The purple formazan crystals were dissolved in 0.5 mL of isopropanol. After 10 min, the plates were read on an automated microplate spectrophotometer (Sunrise, Tecan, Austria) at 570 nm. Assays were performed in triplicate on three independent experiments.

### Annexin V/PI staining assay

For apoptotic cells analysis, SiHa and HeLa cells were treated with various of LicA (10∼50 μM) for 24 hours. The cells were suspended with 100 μl of binding buffer (10 mM HEPES/ NaOH, 140 mM NaCl, 2.5 mM CaCl_2_, pH 7.4) and stained with 5 μl of FITC-conjugated Annexin V and 5 μl of PI (50 μg/ml) for 20 mins at room temperature in dark place and then added 400 μl binding buffer. Apoptotic cells were analyzed via flow cytometry. Samples were analyzed for DNA content with the FACScan flow cytometer (BD Biosciences, San Diego, CA), and relative cell cycle distribution was analyzed using the CellQuest software (Verity Software House, Topsham, ME).

### Acridine orange (AO) staining of autophagic cells

SiHa cells were treated with various concentrations (0, 10, 30, 50 μM) of LicA and 50 μM LicA for 0, 6, 12, and 24 hours. After treatment with LicA, cells were incubated with acridine orange (Sigma) for 30 min. Analysis was performed via fluorescence microscopy using 490-nm band-pass blue excitation filters and a 515-nm long-pass barrier filter. Depending on their acidity, autophagic lysosomes appeared as orange/red fluorescent cytoplasmic vesicles, while cytoplasm and nucleolus were green. To quantitication of autophagy cells, after treatment with LicA, cell were washed with phosphate-buffered saline and stained with 1 μg/mL AO for 15 min at 37°C, AO-stained cells were trypsinized, washed, and quantified by measuring the ratio of red/green fluorescence (FL3/FL1) on a FACSCalibur flow cytometer (Becton, Dickinson and Company) and analyzed using Cell Quest Prosoftware.

### Immunofluorescence assay

For immunofluorescence analysis, cells were grown on glass coverslips and fixed in a 4% paraformaldehyde solution for 10 min at room temperature. After rinsing with PBS, the cells were permeabilized with 0.5% Triton X-100 for 5 min. Following another rinse with PBS, cells were blocked for 15 min at room temperature with 5% BSA buffer. The LC3 antibodies were diluted in 1% BSA and incubated on cells at 37°C with 5% CO_2_ for 2 hours. The coverslips were washed with PBS and placed in rabbit anti-DyLight 488 for 1 hour at room temperature. The cells were washed with PBS and counterstained with DAPI. The cells were examined and photographed by immunofluorescence microscopy.

### Transient tranfection

For transfection, cells were trypsinized and seeded in 6-cm plates at a density of 4 × 10^5^ cells/well. The siRNA-Atg12 (100nM), siRNA-Beclin1 (100 nM), GFP or GFP-LC3 (3 μg) plasmids were introduced plates into the cells using Lipofectamine™ 2000 reagent (Invitrogen) according to the manufacturer's recommendations. The expression vector was transfected 48 hours before treatment with LicA. Cells with green spots were scored under fluorescence microscope.

### Western blot analysis

Cells were lysed using Lysis buffer, and total protein (30 μg) was mixed with Lysis buffer (Bio-Rad Laboratories, Hercules, CA, United States) supplemented with 5% β-mercaptoethanol and heated at 95°C for 5 min followed by 10 min incubation on ice. The sample was then loaded onto a 10 or 12 % SDS-PAGE and subsequently electrotransferred to a PVDF (polyvinylidene difluoride membrane). The membrane was blocked for two hours with 5% non-fat dry milk buffer. After blocking, the membrane was incubated with anti-cleaved-caspase-3, anti-cleaved caspase-9, anti-cleaved PARP, anti-Bcl-2, anti-p-ERK1/2, anti-ERK1/2, anti-p-p38, anti-p38, anti-p-JNK1/2, anti-JNK1/2, anti-p-Akt, anti-Akt, anti-LC3-II, anti-Atg5, anti-Atg7, anti-Atg12, anti-p-mTOR (Ser2448), anti-p-mTOR (Ser2481), anti-mTOR, anti-Raptor, anti-Rictor and anti-β-actin antibody at 1:1000 for overnight at 4°C. After washing, the membrane was incubated with HRP-conjugated anti-mouse (1:10000), anti-goat (1:10000), or anti-rabbit antibody (1:10000) at room temperature for 2 hours. The reaction was visualized using ECL (Pierce) and detected using a Luminescent Image Analyzer LAS-4000 mini.

### Tumor xenograft in nude mice

Tumors were established by injection of 5 × 10^6^ SiHa cells s.c. into the armpit of 4-to 5-week old BALB/c female athymic mice (National Laboratory Animal Center, Taipei, Taiwan). All animal studies were conducted according to the protocols approved by the Institutional Animal Care and Use Committee (IACUC) of Chung Shan Medical University (IACUC Approval No. 1071). Treatments were initiated when tumors reached a mean group size of approximately 85 mm^3^. Tumor volume (cubic millimeters) was measured with calipers, and it was calculated as 0.5236 × L (W)^2^, where W is the width and L is the length of tumor. LicA was administered i.p. to mice 1 times/3 day for 28 days. Tumor volumes were recorded every 7 days until animals were sacrificed.

### Immunohistochemistry

3 mm thick, from representative tissue blocks were cut, deparaffinised with xylene rinse and rehydrated with distilled water through graded alcohol. Immunohistochemical staining was performed on the other four slides using the two-step procedure. The slides were then incubated overnight at 4°C in humidified chambers with human Ki-67 (Abcam; diluted 1:200), cleaved-caspase-3 (Cell signaling; diluted 1:200), cleaved-PARP rabbit monoclonal antibody (Cell signaling; diluted 1:100) and anti-human LC3 rabbit monoclonal antibody (Cell signaling; diluted 1:500) were used. The slides were washed three times in a phosphate buffered solution and further incubated with a secondary antibody for 30 min at room temperature. After washing in phosphate buffered solution, the immunolabeled sections were incubated with biotin-conjugated secondary antibody for 20 min at room temperature, then with peroxidase-conjugated complex (Dako) for 20 min, and finally visualized with 3, 3′-diaminobenzidin and counterstained with hematoxylin, and then examined by light microscopy.

### Statistical analysis

Statistically significant differences were calculated using the Student's *t*-test (Sigma-Stat 2.0, Jandel Scientific, San Rafael, CA, USA). A *p* value < 0.05 or < 0.01 were considered to be statistically significant. Experiments were repeated three times (*n* = 3). Values represent the means ± standard deviation

## RESULTS

### LicA inhibits growth and induces apoptosis in human cervical cancer cells

**Figure 1:**
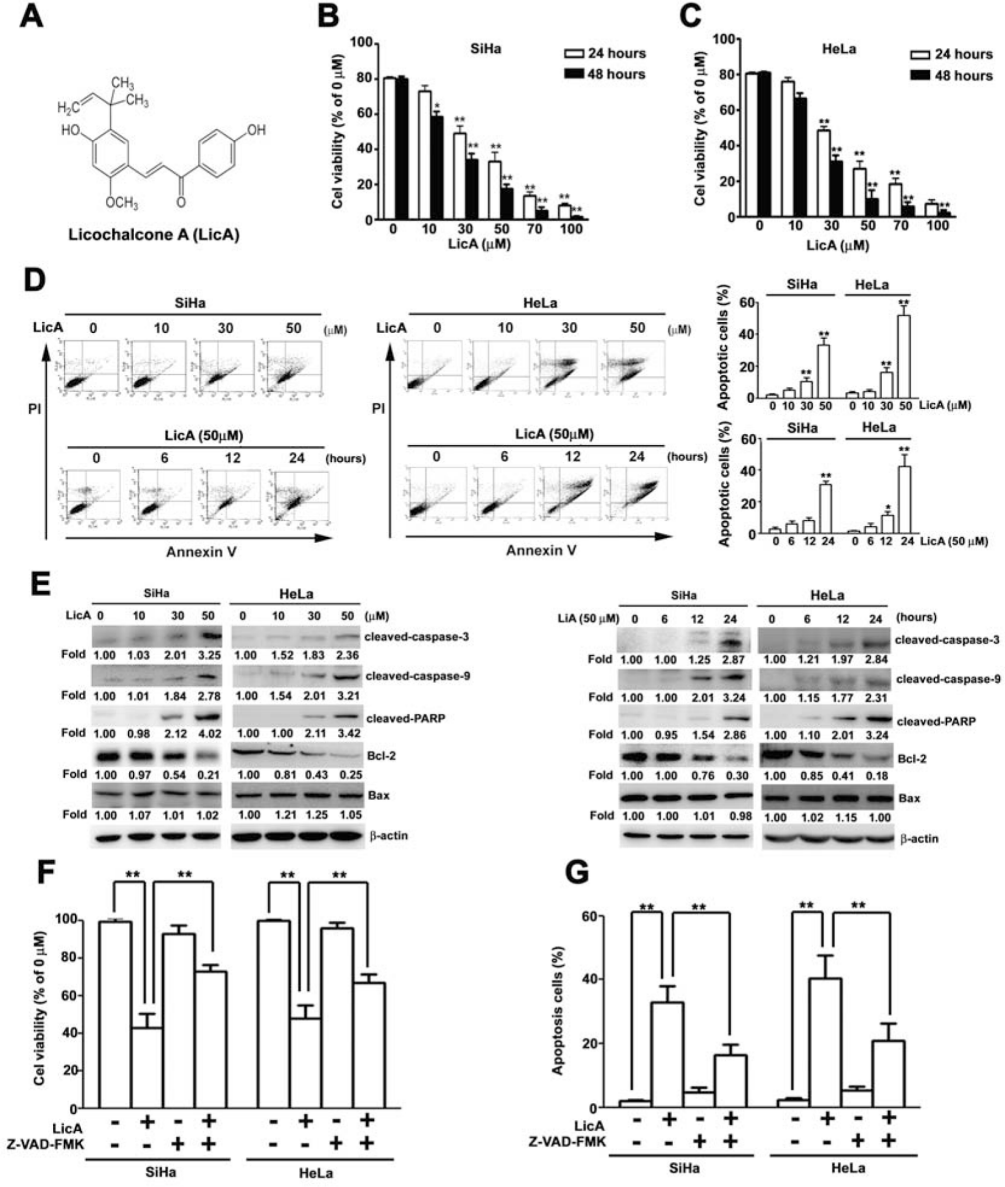
The ability of LicA to induce apoptosis in SiHa cervical cancer cells **A.** The molecular structure of LicA. **B.** SiHa and **C.** HeLa cells were incubated with various concentrations (0, 10, 30, 50, 70, and 100 μM) of LicA for 24 and 48 hours. Cell viability was determined by using an MTT assay. SiHa cells were treated with various concentrations (0∼50 μM) of LicA and 50 μM LicA for 0, 6, 12, and 24 hours. **D.** Then examined by annexin V/PI double stained assay. **E.** Cell lysates were subjected to Western blotting. SiHa and HeLa cells were pretreated with Z-VAD-FMK (25 μM), for 2 hours and then incubated with LicA (50 μM) for 24 hours. **F.** Cell viability was determined by using MTT assay. **G.** The apoptotic cells were measured by flow cytometry. **p < 0.01, untreated cells or LicA plus Z-VAD-FMK versus LicA-treated cells. Data are presented as the mean ± SE of at least three independent experiments.

The chemical structure of Licochalcone A (LicA) is shown in Figure 1A. Prior to investigating the pharmacological potential of LicA for affecting human cervical cancer cell viability, we first assayed the cytotoxicity of LicA by treating SiHa and HeLa cells with LicA at various concentrations (0, 10, 30, and 50 μM) for 24 and 48 hours by using an MTT assay. We found that LicA treatment resulted in significantly decreased viability in SiHa and HeLa cells in a dose- and time-dependent manner, with IC50 values of 42.2 ± 3.5 μM and 48.5 ± 4.2 μM after 24 hours; IC50 values of 32.9 ± 4.2 μM and 40.3±0.8 μM after 48 hours of treatment, respectively (Figure 1B, 1C). Similarly, as shown in Table 1, LicA also inhibited the growth of two other human cervical cancer cell lines (C33A, CaSki and HeLa). Interestingly, LicA was found to be less cytotoxic on two normal cells (HK-2 and WI-38). SiHa and HeLa cells were chosen to represent human cervical cancer for the subsequent studies to elucidate the underlying molecular mechanisms of LicA.

**Table 1:** Summary of cytotoxic efficacies of LicA on cervical cancer cell lines and two normal cell lines

Origin		IC50^a^ ± S.D. (μM)	
		24 hours	48 hours
Cancer cell lines			
SiHa	Human squamous cell carcinoma	42.2 ± 3.5	32.9 ± 4.2
HeLa	Human cervical adenocarcinoma	48.5 ± 4.2	40.3 ± 0.8
CaSki	Human cervical adenocarcinoma	51.2 ± 3.7	43.4 ± 1.5
C33A	Human cervical adenocarcinoma	46.7 ± 4.1	36.8 ± 3.1
Normal cell lines			
HK-2	Human immortalized proximal tubule epithelial cell	88.7 ± 4.5	79.5 ± 5.8
WI-38	Human normal lung fibroblast	90.2 ± 5.1	74.2 ± 3.5

a IC50 is defined at the concentration that results in a 50% decrease in the number of cells compared to that of the control in the absence of LicA. The data shown represent as mean ± SD of three independent experiments.

To determine whether LicA could induce apoptosis in SiHa and HeLa cells, SiHa and HeLa cells were incubated with different concentrations of LicA (0, 10, 30, and 50 μM) and for different durations (0, 6, 12 and 24 hours) with 50 μM LicA. By performing annexin V-FITC/PI double stained assay by flow cytometry, LicA was found to induce apoptosis in SiHa and HeLa cells in a dose- and time-dependent manner (Figure 1D). To further delineate the mechanism by which LicA induced apoptosis in these SiHa and HeLa cells, western blotting assay was performed and revealed that LicA significantly increased the expression of cleaved-caspase-3, cleaved-caspase-9, and cleaved-PARP, while decreasing the expression of Bcl-2 in a dose- and time-dependent manner (Figure 1E). In addition, SiHa and HeLa cells were also pretreated for 2 hours with a pan-caspase inhibitor, Z-VAD-FMK (25 μM), and then incubated with LicA (50 μM) for 24 hours, and the subsequent MTT assays revealed significantly pretreatment with Z-VAD-FMK could effectively attenuate LicA-induced cell viability (Figure 1F) and cell apoptosis (Figure 1G). These results revealed that LicA could induce apoptosis in human SiHa and HeLa cells via the caspase-dependent apoptosis pathway.

### LicA induced autophagy mediated by Beclin-1 and the Atg family in SiHa cells

**Figure 2:**
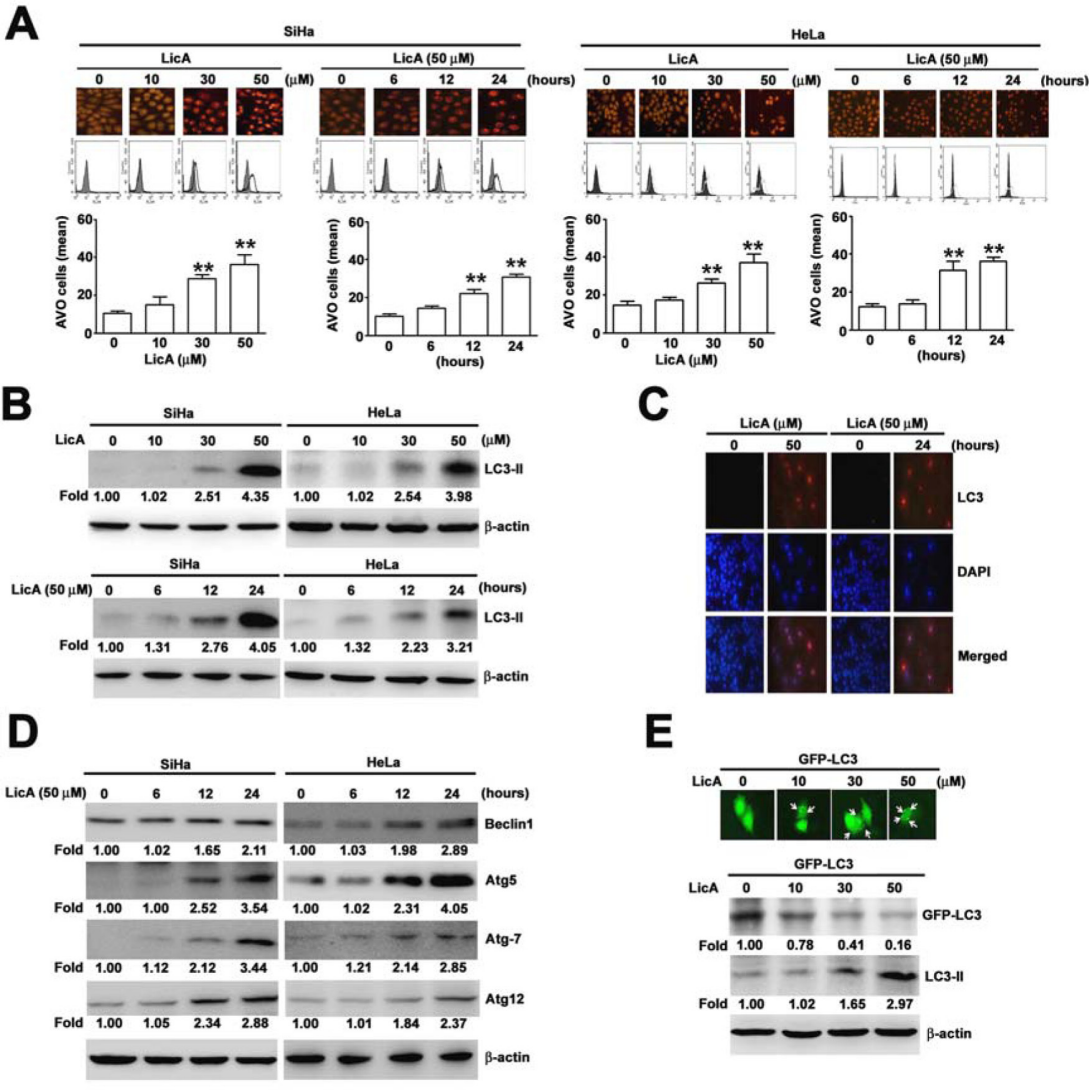
The ability of LicA to induce autophagy in SiHa and HeLa cervical cancer cells **A.** SiHa and HeLa cells were treated with various concentrations (0∼50 μM) of LicA and 50 μM LicA for 0, 6, 12, and 24 hours. **B.** Cell lysates were subjected to Western blotting with anti-LC3-II and anti-β-actin antibodies. **C.** Cells were fixed and immunostained with anti-LC3-II antibody (red), and cell nuclei were counterstained with DAPI reagent. **D.** Effects of LicA on expression of autophagy regulatory proteins. **E.** HeLa cells expressed GFP-LC3 and acidic vesicular organelles were measured by fluorescence microscope and western blotting after the cells were treated with LicA. Data are presented as the mean±SE of at least three independent experiments. **p < 0.01, compared with that of the untreated control (0 μM or 0 hours).

The autophagic pathway begins with the formation of a double-membrane vesicle called the “autophagosome” that engulfs organelles or long-lived proteins and then matures into an acidic single-membrane autophagosome that fuses with a lysosome to become the “autolysosome”. This process is known to be accompanied by an increase in the acidity of the lumen, followed by the development of acidic vesicular organelles (AVOs) [26]. AVO reagent staining showed that the relative fluorescence intensity of SiHa and HeLa cells was increased in a dose- and time-dependent manner (Figure 2A, upper). The quantification of AVO cells by flow cytometry assay (Figure 2A, down) also indicated the occurrence of autophagy. The amount of LC3-II cleaved product is correlated with the extent of autophagosome formation and detection of autophagic activity [27, 28]. To elucidate whether LicA could induce autophagy in SiHa cells, SiHa cells were incubated with various concentrations of LicA (0, 10, 30, and 50 μM) and for different durations (0, 6, 12 and 24 hours) with 50 μM LicA. According to Western blotting assays, we found that SiHa cells treated with increasing concentrations of LicA resulted in dose-dependent increased expression of LC3-II (Figure 2B). Similarly, confocal fluorescence microscopy indicated that LicA increased the formation of LC3-II in SiHa cells treated with 50 μM LicA or incubated in 50 μM LicA for 24 hours (Figure 2C). In addition, a trend of increased expression of other autophagy-associated proteins such as Beclin1, Atg5, and Atg7 with increased expression of Atg12 was also observed in LicA treated cells (Figure 2D). When performing a GFP-LC3 transfection study, we used the HeLa cervical cancer cell line instead of the SiHa cell line because doing so was more efficient. After transfection with GFP-LC3 and treatment with LicA for 48 hours, cytoplasmic LC3II formation was observed to occur in a dose-dependent manner in HeLa cells treated with LicA (Figure 2E, upper). Moreover, Western blotting assays indicated that the expression levels of LC3-II were increased in a dose-dependent manner (Figure 2E, down). Together, these results supported the idea that LicA induced autophagy in SiHa and HeLa cells.

### Inhibition of autophagy enhances LicA-induced apoptosis

**Figure 3:**
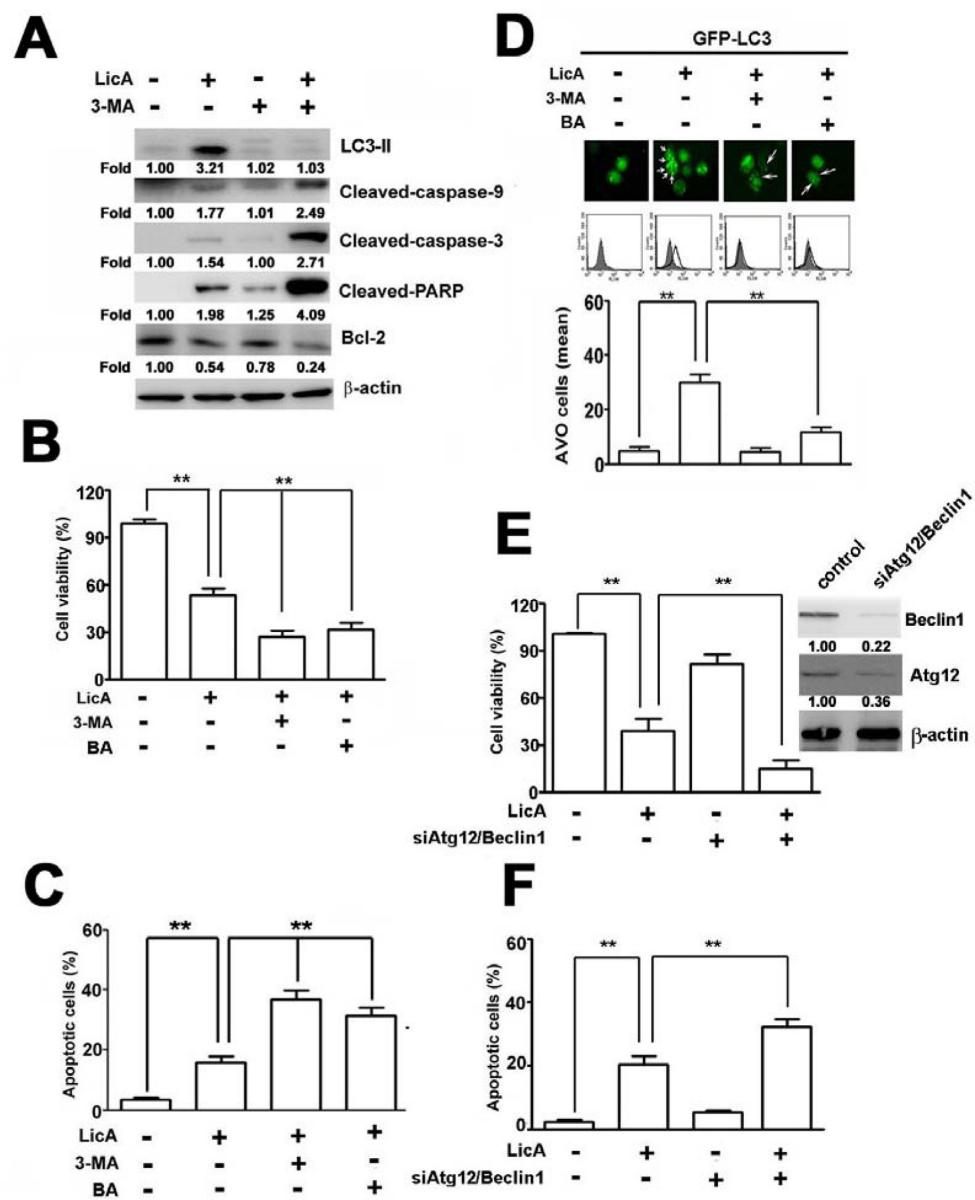
Autophagy decreased LicA-induced apoptosis in SiHa cervical cancer cells SiHa cells were pretreated with an autophagy inhibitor, 3-MA (10 mM) and bafilomycin A (BA; 10 nM) for 2 hours and then incubated with LicA (50 μM) for 24 hours. **A.** Cell lysates were subjected to Western blotting. **B.** Cell viability was determined by using MTT assay. **C.** The apoptotic cells were measured by flow cytometry. **D.** Cells expressed production of acidic vesicular organelles and quantification were examined by fluorescence microscope and flow cytometry. **E.** SiHa cells treated with the combination of LicA and siAtg12/siBeclin-1 duplexes. Cell viability was assayed or **F.** annexin V-positive cells were quantitatively analyzed. **p < 0.01, untreated cells or LicA plus 3-MA, BA, or siAtg12/siBeclin1 versus LicA-treated cells. Data are presented as the mean ± SE of at least three independent experiments.

As described above, we found that SiHa cells treated with LicA exhibited increased apoptosis and autophagy. To determine the inter-relationship between apoptosis and autophagy after treating SiHa cells with LicA, we found that treating SiHa cells with LicA and 10 mM 3-MA (an inhibitor of autophagy) increased the expression of cleaved caspase-9, cleaved caspase-3, and cleaved PARP, and decreased the expression of Bcl-2 more than treating SiHa cells with LicA alone (Figure 3A). Here, we used bafilomycin A (BA), an autophagy-lysosomal inhibitor [29]. MTT assays showed that the apoptotic effect of LicA on SiHa cells was enhanced when LicA was combined with 3-MA or BA in comparison to treatment with LicA alone (Figure 3B). In addition, annexin V-FITC/PI double stained assays revealed that treatment of SiHa cells with LicA and 3-MA or BA resulted in a significantly greater number of apoptotic cells than treatment with LicA alone (Figure 3C). Moreover, after transfection with GFP-LC3 for 48 hours, then treatment with LicA for another 24 hours, cytoplasmic LC3II formation was observed in HeLa cells treated with LicA (Figure 3D, upper), and subsequent treatment with LicA and 3-MA (10 mM) or BA (10 nM) significantly reduced the formation of cytoplasmic LC3-II and acidic autophagic vacuoles (Figure 3D, down). In addition, SiHa cells after knockdown of Atg12/Beclin1 for 48 hours, as subsequent treatment with LicA for another 24 hours resulted in remarkably increased cell apoptosis (Figure 3E, 3F). These results indicated that suppression of autophagy could enhanced the LicA-induced apoptosis.

### Inhibition of autophagy enhances LicA-induced apoptosis via the PI3K/Akt signaling pathway

**Figure 4:**
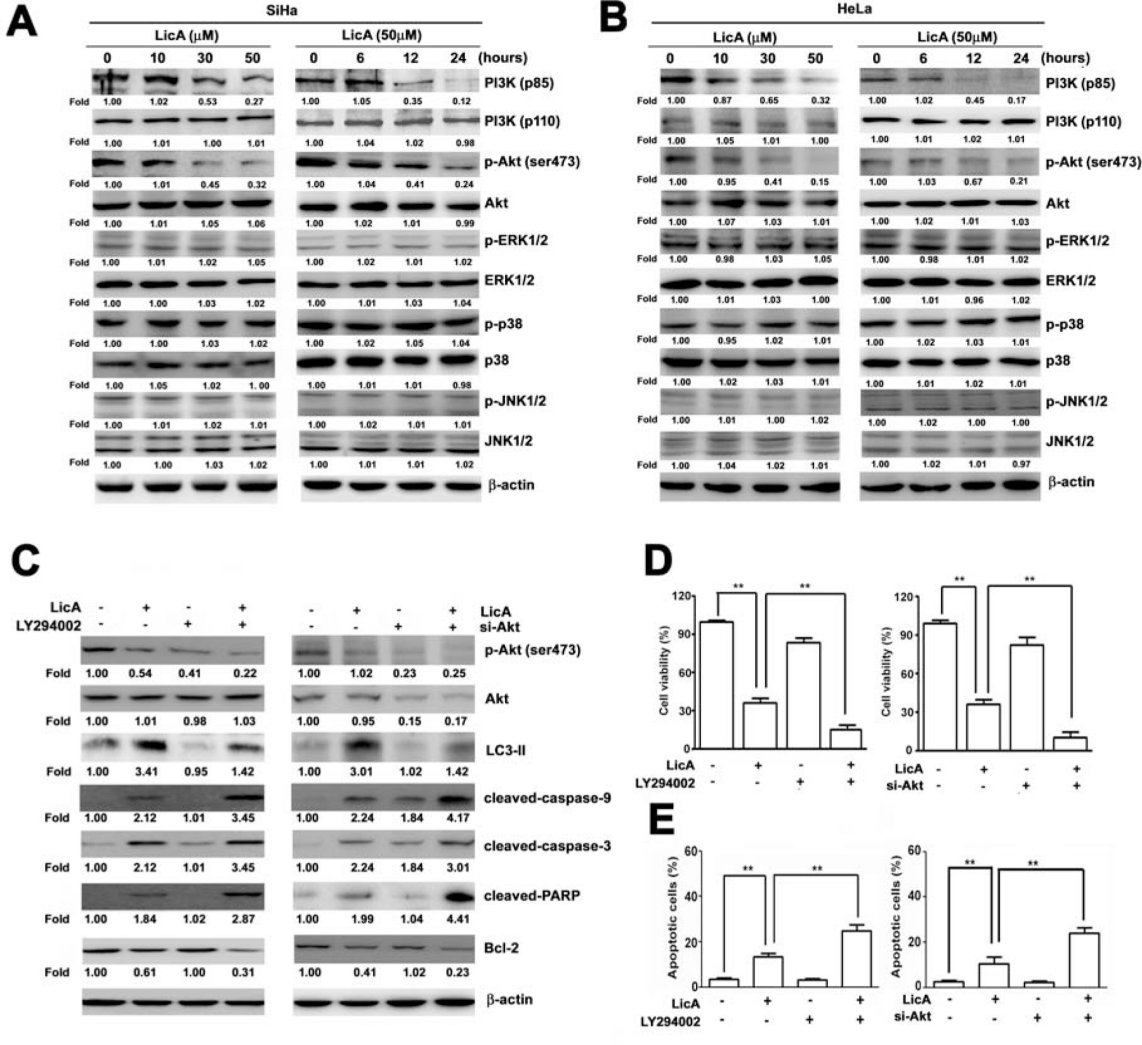
LicA induced apoptosis via the PI3K/Akt signaling pathway in SiHa and HeLa cells SiHa and HeLa cells were treated with various concentrations (0∼50 μM) of LicA and 50 μM LicA for 0, 6, 12, and 24 hours. **A.** Cell lysates were subjected to Western blotting. **B.** SiHa and HeLa cells were treated with LicA in the absence or presence of LY294002 (15 μM) and siAkt (100 nM) by Western blotting. **C.** Cell viability was measured by MTT assay and **D.** the proportion of annexin V-positive cells was analyzed by flow cytometry. **p < 0.01, untreated cells or LicA plus LY294002 or siAkt versus LicA-treated cells. Data are presented as the mean ± SE of at least three independent experiments.

It has previously been shown that the MAPK, PI3K/Akt and mTOR signaling pathway is involved in the molecular biological mechanisms by which autophagy occurs [30]. After analyzing western blotting assay results to investigate whether the effect of LicA on SiHa and HeLa cells occurred via this pathway, we found that treated with LicA showed decreasing expression of PI3K (p85) and phosphorylated Akt (ser473) in a time- and dose-dependent manner in both SiHa (Figure 4A) and HeLa cells (Figure 4B), but not of phosphorylated-ERK1/2, phosphorylated-p38, or phosphorylated-JNK1/2. To confirm that the LicA-induced autophagy occurred via the PI3/Akt signaling pathway, we employed LY294002 or si-Akt to silence Akt, treating SiHa cells with LicA and LY294002 or si-Akt significantly increased the expression of cleaved-caspase-9, cleaved-caspase-3, and cleaved-PARP, and decreased the expression of Bcl-2 and LC3-II in comparison to than treatment with LicA alone (Figure 4C). The MTT assay results showed that the enhanced apoptotic effects of LicA on SiHa cells were enhanced when treatment with LicA was combined with LY294002 or si-Akt in comparison to treatment with LicA alone (Figure 4D). In addition, annexin V-FITC/PI double stained assays revealed that LicA combined with LY294002 or si-Akt resulted in a significantly greater number of apoptotic cells than treatment with LicA alone (Figure 4E). These results indicated that LicA induced autophagy and apoptosis are indeed via inhibition of the phosphorylation of PI3K/Akt pathway.

### Inhibition of autophagy enhances LicA-induced apoptosis via inhibition of mTOR phosphorylation

**Figure 5:**
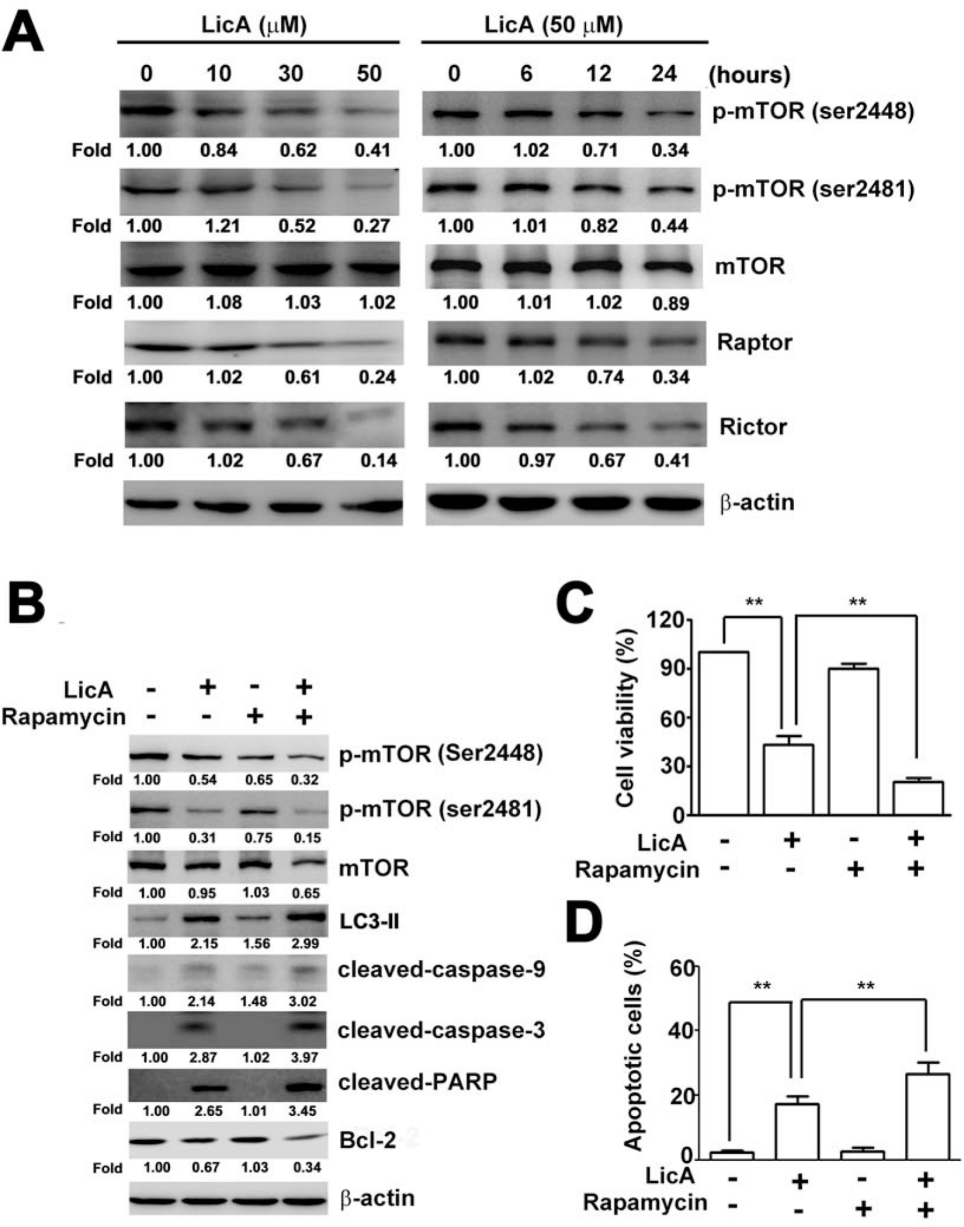
LicA-induced apoptosis and autophagy via the mTOR pathway SiHa cells were treated with various concentrations (0∼50 μM) of LicA and 50 μM LicA for 0, 6, 12, and 24 hours. **A.** Cell lysates were subjected to Western blotting. **B.** SiHa cells were treated with LicA in the absence or presence of rapamycin (100 nM) by Western blotting. **C.** Cell viability was measured by MTT assay and **D.** the proportion of annexin V-positive cells was analyzed by flow cytometry. **p < 0.01, untreated cells or LicA plus rapamycin versus LicA-treated cells. Data are presented as the mean ± SE of at least three independent experiments.

It is well-known that the mTOR pathway functions as an autophagy regulator under starvation or other cellular stress conditions [31]. We detected the expression of phosphor-mTOR (ser2448), phosphor-mTOR (ser2481), mTOR, Raptor and Rictor in SiHa cells treated with different concentrations of LicA (0, 10, 30, and 50 μM) and different durations (0, 6, 12 and 24 hours) with 50 μM LicA. As shown in Figure 5A, Western blotting assays revealed that LicA significantly decreased the expression of phosphor-mTOR (ser2448), phosphor-mTOR (ser2481), Raptor and Rictor in a dose- and time-dependent manner. We next raised the question of whether inhibition of mTOR (autophagy regulator) expression affects LicA-induced apoptosis. Western blotting analysis then revealed significantly increased LC3-II, phosphor-mTOR (ser2448), phosphor-mTOR (ser2481), cleaved-caspase-3, cleaved-caspase-9, and cleaved-PARP expression, and decreased Bcl-2 expression in SiHa cells treated with rapamycin (an mTOR inhibitor) and LicA compared with cells treated with LicA alone (Figure 5B). In addition, combined treatment with rapamycin and LicA significantly inhibited cell viability (Figure 5C) as well as increased the population of annexin V-positive cells (Figure 5D) compared with LicA treatment alone. These results suggest that LicA induces inhibition of the mTOR signaling pathway and thus results in the subsequent induction of autophagy and apoptosis.

### LicA inhibits growth of SiHa cell xenografts *in vivo*

**Figure 6:**
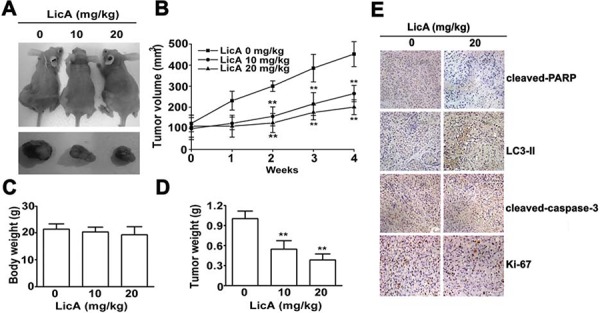
LicA inhibited the tumor progression of cervical cancer *in vivo* **A.** Nude mice inoculated with SiHa cervical cancer cells were fed with 0, 10, or 20 mg/kg LicA continuously until study termination. **B.** The growth of tumors was monitored in terms of tumor volume every week. The weights of the mice **C.** and the tumors **D.** were determined. **E.** IHC stainings of cleaved-caspase-3, cleaved-PARP, LC3-II and Ki-67 in untreated and LicA-treated SiHa xenografts. **p < 0.01, compared with that of the untreated control (DMSO).

LicA could induce apoptosis and autophagy in cervical cancer cells *in vitro*, it was necessary to test whether LicA could inhibit the progression of cervical cancer cells in vivo. To test the effects of LicA on the progression of human cervical cancer cells, nude mice inoculated with SiHa cervical cancer cells were continuously fed with varying concentrations of LicA (0 mg/kg, 10 mg/kg, and 20 mg/kg) until study termination. After the administration of LicA at 10 and 20 mg/kg in mice with SiHa tumor xenografts for 28 days, the growth of tumors was significantly reduced by 43.8% and 61.2%, respectively (Figure 6A and 6B), compared with DMSO controls. Ultimately, there were no significant differences in mean body weight between the control group and the treatment groups, which indicated that there were no adverse effects from feeding nude mice with LicA (Figure 6C). In addition, the tumor weights of SiHa tumor xenografts were significantly lower in the LicA-treated groups than in the control group (Figure 6D). Then IHC staining of cleaved-PARP, cleaved-caspase-3, LC-II, and Ki-67 were performed to investigate the apoptosis, and autophagy and proliferation in the xenograft tumor sections. We found upregulation of cleaved-PARP, cleaved-caspase-3 and LC3-II, and downregulation of proliferation marker Ki-67 of the LicA-treated SiHa groups (Figure 6E). Collectively, these results suggested that LicA inhibits tumor growth in cervical cancer cells

## DISCUSSION

**Figure 7:**
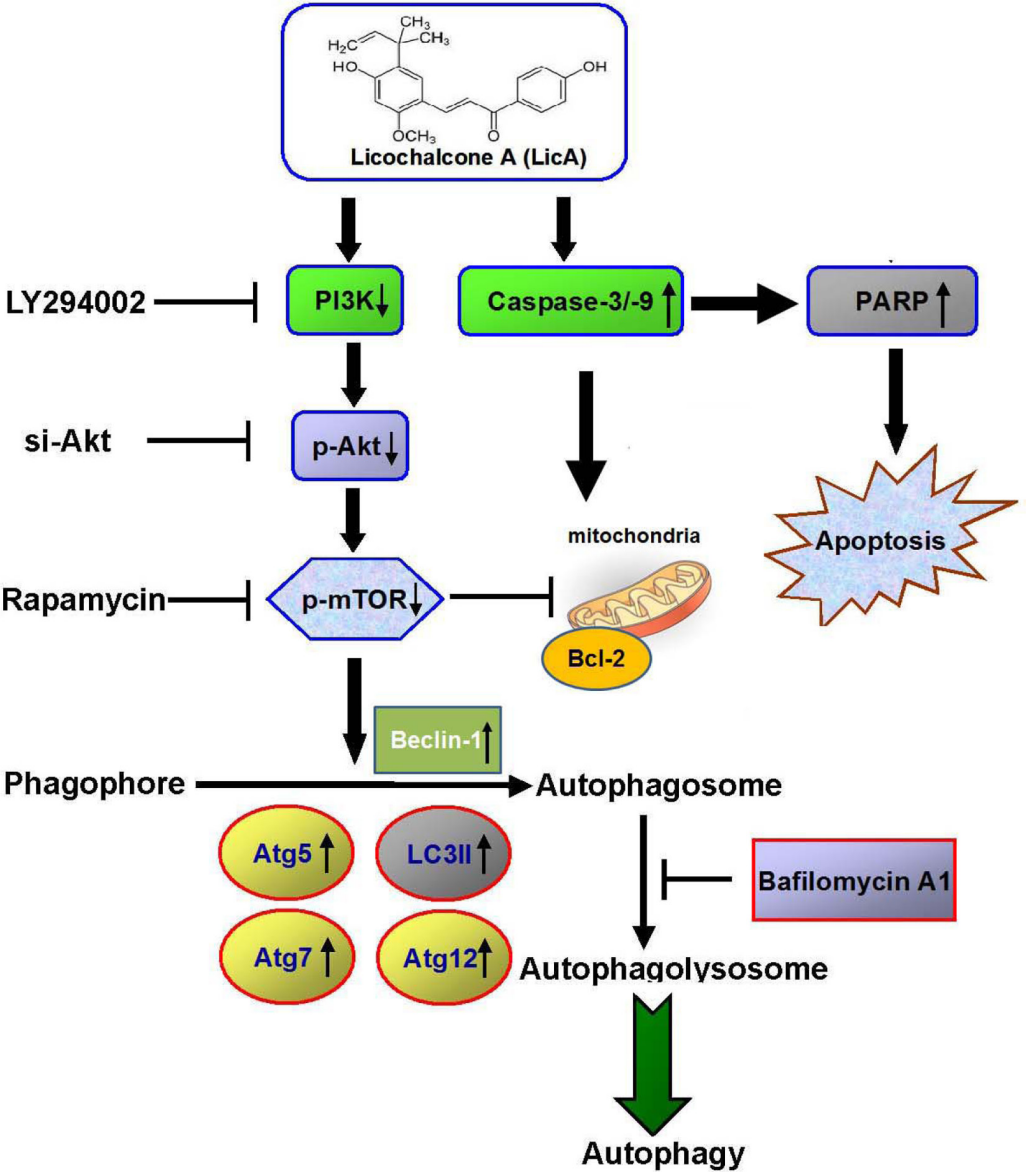
Overview of pathways for LicA-inhibited the phosphorylation of PI3K-Akt-mTOR axis and autophagy inhibition enhances LicA-induced apoptosis in human cervical cancer cells

It had been known that cervical cancer leaded the third-most cancer among women worldwide, resulting in an annual 250,000 deaths [32]. Its incidence in developed countries, however, has decreased by around 70% over the past 50 years due to improved screening methods in cervical cytology [33]. Recently, many naturally grown plant agents have been reported to induce autophagy, but the effects of altered autophagy are inconsistent, because it can either protect cells from apoptosis or promote cell death [34, 35]. One of the flavonoids, Licochalcone A, which is the root of Glycyrrhiza inflate, has previously been reported to have effects on cell cycle progression, such as inhibiting the proliferation of vascular smooth muscle cells by blocking G1 to S phase progression [21], arresting progression at the G2/M phase transition in prostate cancer [36] and gastric cancer [37], and inhibiting the invasion and migration of hepatocellular carcinoma cells by inhibiting uPA expression [22]. Some researchers have also reported that isoliquiritigenin, which is a constituent of licorice (Glycyrrhiza inflate), can decrease cell viability, induce the accumulation of cells in the G2/M phase, and enhance apoptosis, in addition to causing changes in Bax and Bak expression, decreasing the expression of Bcl-2 and Bid proform, triggering dissipation of the mitochondrial membrane potential, and releasing cytochrome c to the cytosol followed by activation of the caspase cascade with cleavage of caspase-9, caspase-3, and PARP [38, 39]. This sequence of events is strongly supported by the results of the current study. First, we found that LicA is a potent inhibitor of cervical cancer cells *in vitro*. Second, we found that LicA induces apoptosis and autophagy in cervical cancer cells. Third, the selective autophagy protein Beclin 1, Atg5, Atg7, Atg12 and autophagosome-associated form (LC3-II) was demonstrated by western blotting. Fourth, the results showed that LicA induced its effects on autophagy and apoptosis in cervical cancer cells via inhibition of the PI3K/Akt/mTOR signaling pathway (Figure 7). Finally, xenograft tumor growth was inhibited by LicA. Overall, the results indicate that LicA activated the autophagic and apoptotic processes in vitro in cancer cells and *in vivo* in tumor xenograft models.

Autophagy, which is characterized by the accumulation of autophagic vacuoles, no forming of apoptotic bodies, no condensing of chromosomes, and caspase independence, is a process of physiological cell death that occurs during development. Another type of programmed cell death, apoptosis, involves the early collapse of cytoskeletal elements but preservation of organelles until late in the process and is caspase-dependent [40]. Although the role of the autophagic process in antitumor therapy has not been clearly elucidated, many studies have demonstrated that these two types of cell death are predominantly distinctive but that cross-talk also occurs between them [41–43]. In this study, we found that LicA-treated SiHa cells presented with more annexin V-positive cells and expressed enhanced activities in terms of cleaved caspase-3, cleaved caspase-9, cleaved PARP, but less Bcl-2, all of which indicated the ability of LicA to induce apoptosis. We considered the possibility that LicA could inhibit the growth of cervical cancer cells by concomitantly inducing apoptosis and autophagy. Studies had previously shown that after treatment with antitumor drugs, some cancer cells undergo autophagy as a temporary survival mechanism, and the suppression of autophagy leads to apoptosis, thus enhancing antitumor effects; however, some treatments also result in the induction of autophagic cell death or both apoptosis and autophagy. Xu et al. proposed that inhibiting autophagy by 3-MA or chloroquine in cervical cancer enhanced cellular apoptosis [41]. Sun et al. found that over-expression of Beclin-1, which is an autophagy-related gene, may enhance the activity of apoptosis in cervical cancer cells [44]. Hou et al. reported that fucoxanthin induces HeLa cervical cell autophagy but not apoptosis, even with 3-MA pretreatment [45]. Hsu et al. found increased activities of apoptosis, autophagy, and anti-proliferation after treating cervical cancer cells with resveratrol [40]. So, it seems that there is complex relationship between autophagy and apoptosis when cells are treated with different substances. However, the exact role of autophagy in cancer treatment, and the question of whether it can protect cells from the cytotoxic effects of anticancer drugs by blocking apoptosis or by killing cells as an alternate pathway to cell death is still controversial [46]. In this study, we found that for cervical cancer cells treated with LicA, autophagy might play a role in fine tuning the cellular apoptotic ability.

The autophagic genes Beclin-1, Atg5, Atg7, Atg12, and LC3 are responsible for autophagosome biogenesis [47]. Atg7 is reported to be essential for Atg12 conjugation and LC3 modification systems, and the conversion of LC3-I to LC3-II correlates with autophagosome formation [48]. Recent studies have also shown that the expression of Atg5, Atg7, and Atg12 proteins plays a role in autophagic/apoptotic cell death and gastrointestinal cancer pathogenesis [49, 50]. Patients with increased Beclin1 expression in their cancer cells exhibit longer survival [51], and so Beclin1 expression has been postulated to act as a tumor suppressor [52]. In this study, we demonstrated by western blotting that LicA increases the expression levels of Atg5, Atg7, Atg12, Beclin1, and LC3-II in a time-dependent manner in SiHa cells. This finding is consistent with a recent report that dendropanoxide (DP), which comes from the leaves and stem of Dendropanax morbiferus H.Lév, induced a time-dependent accumulation of Atg7, Beclin1, and LC3-II in osteosarcoma cells, and that treatment with U0126, 3-MA, and wortmannin significantly decreased DP-induced autophagy that was accompanied by increased apoptosis [53]. Another study, reported that aristolochic acid I (AAI) induced autophagy and apoptosis in renal tubular epithelial cells, and that the knockdown of Beclin1 or Atg7 not only decreased the LC3-II formation induced by AAI, but also induced apoptosis [54]. Our study demonstrates that gene silencing of Atg12 and Beclin1, or cotreatment of the cervical cancer with 3-MA or BA, inhibits LicA-induced autophagy. Therefore, LicA-induced autophagy may serve as a protective mechanism against LicA-induced apoptosis.

Bcl-2 was reported to act as an anti-autophagy protein via its inhibitory interaction with Beclin-1 in various cancer cells [55, 56]. In human leukemic HL60 cells, down-regulation of Bcl-2 was induced autophagy-related cell death [57]. Tetrapeptide ICE inhibitor induced loss of the mitochondrial membrane potential (MMP) and promoted cell death, which it may be attributed to the decreased expression of Bcl-2 in [58]. Recently, it has been reported that silibinin induced the LC3-II expression which closely correlate with the number of autophagosomes, as well as Atg12-Atg5 formation were increased, elevated expression of Beclin1 which was accompanied by a decreased level of Bcl-2 in MCF7 breast cancer cells [59]. Licorice extract and LicA may have induced an imbalance between Bcl-2 and Beclin1 levels in LNCaP cells, leading to the promotion of autophagy [24]. Consistent with the our finding of enhanced LC3-II and Atg-related proteins expression by LicA-treated cervical cancer cells, Therefore, it is possible that LicA-inhibited autophagy was accompanied by apoptosis, and the interplay between apoptosis and autophagy occurred, which warrants further investigation.

Many pieces of evidence suggest that the cross-talk between apoptosis and autophagy is made especially complicated by the fact that they share many common regulatory molecules, such as Bcl-2 and the PI3K/Akt/mTOR signaling pathway [60]. It is well known that the PI3K/Akt pathway serves as a critical signaling axis in cell growth, proliferation, and cell survival, but intriguingly, several lines of evidence suggest that this pathway could also provide a pro-death signal, especially for necrotic cell death [61]. For instance, it has been reported that the activation of PI3K and Akt sensitized the necrotic cell death in response to glucose deprivation or hypoxia in rat cardiomyocytes [62] and mouse Lewis lung carcinoma cells, respectively [63]. LicA could induces ROS-mediated MAPKs activation, inhibit PI3K/AKT pathway, and lead to gastric cancer cell apoptosis [64]. The inhibitory effects of LicA on PI3K/Akt are consistent with the prevention of phosphorylation of Akt downstream targets. mTOR is major downstream target of Akt, and inhibition of the PI3K/Akt/mTOR pathway has been shown to initiate autophagy and apoptosis [65, 66]. Here, we observed the role of the mTOR pathway in autophagy pathway, because some studies indicated that inhibition of the Akt-mTOR pathway was consistently associated with triggering autophagy in tumor cells [67]. It have been reported that phosphorylation of Akt at Ser473 is associated with resistance to apoptosis by controlling subcellular localization of pro-apoptotic proteins [68]. Our data showed that the LicA decrease in Akt at Ser473 phosphorylation in SiHa and HeLa cells. It can be suggested that phosphorylation of Akt at Ser473 may play a protective role in LicA-induced apoptosis. Moreover, pretreatment of mTOR inhibitor (rapamycin) not only reinforces the apoptotic-inducing activity and antitumor capacity of LicA, but also probably autophagy induction. Therefore, our study demonstrated that mTOR might act as an important factor governing the cross talk between apoptosis and autophagy in human cervical cancer cells. Notwithstanding, the role of mTOR in manipulating LicA-induced cell apoptosis and autophagy appears complicated and needs to be further explored.

In the present study, we found that LicA substantially increased the levels of LC3-II, in addition to increasing caspase-3, caspase-9, and PARP cleavage, in cervical cancer cells, all of which suggested that LicA induces both apoptosis and autophagy in these cells. We also found, via an *in vivo* study, that LicA inhibited the growth of xenografts of cervical cancer in nude mice. The phenomenon of enhanced apoptotic ability cause by combining LicA treatment with an autophagy inhibitor and PI3K suggested that LicA-induced autophagy prevents cervical cancer cells from undergoing notable apoptosis. Collectively, we conclude that LicA induced apoptosis, inhibited PI3K/Akt/mTOR signaling, and resulted in autophagy, highlighting a basic mechanism accounting for the anticancer activity of LicA and a potential strategy to enhance the anticancer efficacy of LicA by preventing autophagy.
